# Integrative competing endogenous RNA network analyses identify novel lncRNA and genes implicated in metastatic breast cancer

**DOI:** 10.1038/s41598-023-29585-x

**Published:** 2023-02-10

**Authors:** Dulari K. Jayarathna, Miguel E. Rentería, Jyotsna Batra, Neha S. Gandhi

**Affiliations:** 1 Centre for Genomics and Personalised Health, School of Chemistry and Physics, Queensland University of Technology, 2 George Street, Brisbane, QLD 4000 Australia; 2 Department of Genetics and Computational Biology, QIMR Berghofer Medical Research Institute, Brisbane, QLD 4006 Australia; 3 School of Biomedical Sciences, Faculty of Health, Queensland University of Technology, Kelvin Grove, Brisbane, QLD 4059 Australia; 4 Translational Research Institute, 37 Kent Street, Woolloongabba, QLD 4102 Australia

**Keywords:** Computational biology and bioinformatics, Genetics

## Abstract

Competing endogenous RNAs (ceRNAs) have gained attention in cancer research owing to their involvement in microRNA-mediated gene regulation. Previous studies have identified ceRNA networks of individual cancers. Nevertheless, none of these studies has investigated different cancer stages. We identify stage-specific ceRNAs in breast cancer using the cancer genome atlas data. Moreover, we investigate the molecular functions and prognostic ability of ceRNAs involved in stage I–IV networks. We identified differentially expressed candidate ceRNAs using edgeR and limma R packages. A three-step analysis was used to identify statistically significant ceRNAs of each stage. Survival analysis and functional enrichment analysis were conducted to identify molecular functions and prognostic ability. We found five genes and one long non-coding RNA unique to the stage IV ceRNA network. These genes have been described in previous breast cancer studies. Genes acted as ceRNAs are enriched in cancer-associated pathways. Two, three, and three microRNAs from stages I, II, and III were prognostic from the Kaplan–Meier survival analysis. Our results reveal a set of unique ceRNAs in metastatic breast cancer. Further experimental work is required to evaluate their role in metastasis. Moreover, identifying stage-specific ceRNAs will improve the understanding of personalised therapeutics in breast cancer.

## Introduction

Breast cancer (BC) is currently diagnosed in 1 in 8 Australian women over their lifetime, making it as the primary cause of female cancer-associated deaths in Australia. Decades of studies have identified candidate prognostic biomarkers for BC. Recent bioinformatic and experimental studies have found that microRNAs (miRNAs) can act as biomarkers in BC as they play a crucial role in transcriptional and post-transcriptional gene regulation. miRNAs belong to a group of small non-coding RNAs with 19–25 nucleotides in length. According to conventional RNA logic, miRNAs inhibit/degrade gene expression binding with miRNA response elements (MREs) of messenger RNAs (mRNAs)^1^. Salmena, Poliseno^2^ introduced the competing endogenous RNA (ceRNA) hypothesis revealing the bi-directional regulation mechanism of miRNAs. The ceRNA logic explains that non-coding RNA transcripts such as long non-coding RNAs (lncRNAs) with similar MREs can also bind with the relevant miRNAs modulating gene regulation and protein networks. Due to these reasons, ceRNAs have gained considerable attention in cancer studies.

Previous bioinformatics and experimental studies have identified many ceRNAs associated with BC risk^3^. Apart from generalising results for BC incidents, different cancer stages can also severely impact response to therapy and mortality. In BC, stage I refers to a tumour or small size confined to the breast; stage II explains the disease that has locally advanced beyond the breast; stage III describes BC has spread to the neighbouring organs, and stage IV refers to distant metastatic disease^4^. The early stages, I and II, are considered treatable compared to advanced stages, III and IV, that require more radical and active treatment strategies^5^. Therefore, identifying stage-specific biomarkers in BC will significantly contribute to understanding BC biology under different pathological states.

In this study, we performed a ceRNA network analysis to identify stage-specific ceRNAs in BC, and this approach can be applied for any disease of interest in the future. The identified ceRNAs were further studied using two downstream analyses to identify their molecular functions and prognostic ability.

## Materials and methods

### Patients and samples collection

The expression data (RNA-seq and miRNA-seq) and clinical data of BC were collected from the cancer genome atlas (TCGA) that contains 1091 cases and 113 controls. The HTSeq-counts data of RNA-seq (including protein-coding and long non-coding) and isoform quantification data of miRNA-seq (in BC) were downloaded to a local computing server using the genomics data commons (GDC) data portal^6^. For selecting miRNA-seq data for individuals, a manifest file was generated using the GDC Data Portal. Then, the GDC data transfer tool was used to transfer data files listed in the manifest file. The differential expression analysis produced the "group" variable to identify differentially expressed protein-coding genes and long non-coding RNAs.

### Differential expression analysis

Firstly, we removed TCGA BC samples with duplicated sample IDs. Then samples that are neither solid tissue normal nor primary tumour were removed as we compared primary tumour and healthy samples in the differential expression analysis. First, we performed the counts per million (CPM) normalisation to correct sample library size differences. The low-expressed genes that log (CPM) < 1 in more than 50% of the samples were removed before the differential expression analysis^7^. Ignoring low-expressed genes improves the total count of differentially expressed genes and enhances sensitivity and precision. Raw counts expression data were re-normalised using the TMM (trimmed mean of M values) method implemented in the edgeR (3.40.0) R package (https://bioconductor.org/packages/release/bioc/html/edgeR.html) to compare expression levels between samples (excluding low-expressed genes)^8^. The normalised data were transformed into a standard scale using the voom function in the limma (3.54.0, https://bioconductor.org/packages/release/bioc/html/limma.html) (linear modelling for microarrays) R package^9^. Previous RNA-seq data analysis-related works have recommended this hybrid technique, TMM normalisation with voom transformation, due to its better performance in data preprocessing^10,11^. Moreover, Oshlack et al. have shown TMM normalisation is robust and outperforms library size normalisation^12^. In differential expression analysis, linear models were fitted for each gene using the "lmFit" function implemented in the limma (3.54.0, https://bioconductor.org/packages/release/bioc/html/limma.html) R package^9^. Then eBayes moderation was applied using information across all the genes to obtain more precise estimates of gene-wise variability. Four differential expression analyses were conducted for stage I to IV-control comparisons. The cancer stage was determined using the "pathologic stage" as it provides more accurate information combining results from clinical examinations and surgeries. We gathered 181 (19% basal-like, 5% HER2+, 62% luminal A, 13% luminal B, and 1% normal-like), 619 (21% basal-like, 13% HER2+, 40% luminal A, 25% luminal B, and 1% normal-like), 247 (12% basal-like, 16% HER2+, 40% luminal A, 29% luminal B, and 3% normal-like), and 20 (17% basal-like, 16% HER2+, 25% luminal A, and 42% luminal B) samples for stages I, II, III, and IV, respectively. In each stage-specific expression analysis, differentially expressed mRNAs, lncRNAs, and miRNAs were defined at |log2-fold change (FC)|> 1 and Benjamini–Hochberg (BH)-adjusted p value (default in limma package) < 0.05^13^.

### Competing endogenous RNA network analysis

The differentially expressed mRNAs, lncRNAs, and miRNAs in each cancer stage were applied in the stage-specific ceRNA network analysis. The ceRNA network analysis consists of three main steps: (1) identifying lncRNA-mRNA pairs that share the significant number of miRNAs, (2) selecting positively correlated lncRNA-mRNA pairs, and (3) jointly estimating the significance of multiple miRNAs in lncRNA-mRNA pairs. These three steps are described in detail in previous ceRNA papers^14,15^. The mRNA-miRNA and lncRNA-miRNA interactions are required to perform steps i and iii. We used miRcode and starBase databases for miRNA-target predictions^16,17^. The miRcode database facilitates mRNA-miRNA and lncRNA-miRNA target predictions using a broad searchable map that contains 10,419 lncRNAs. The starBase includes miRNA-mRNA interactions predicted by analysing 108 CLIP-seq datasets. Steps i and ii were performed using the hypergeometric test and the Pearson correlation test, respectively. These two testing methods have been implemented in the GDCRNATools (1.18.0, https://bioconductor.org/packages/release/bioc/html/GDCRNATools.html) R/Bioconductor package^18^. The third step, multiple sensitivity correlation (mscor) analysis, was executed using the SPONGE (1.20.0, https://bioconductor.org/packages/release/bioc/html/SPONGE.html) (sparse partial correlation on gene expression) R/Bioconductor package^19^. The significant ceRNA interactions were filtered by three user-defined thresholds, (1) false discovery rate (FDR) < 0.01 in hypergeometric test, (2) Pearson correlation coefficient > 0.40, and (3) the adjusted p value of mscor in SPONGE method < 0.05. The resulting lncRNA-miRNA-mRNA associations in each BC stage were combined into a single column, as "<lncRNA gene ensemble ID>_<gene ensemble ID>_<miRNA name>". Then set of values in each BC stage were applied into a four-sets (for four stages) Venn diagram representation.

### Functional enrichment analysis

A total of unique 47 aberrantly expressed genes (25 from stage I, 42 of stage II, 40 of stage III, and 47 of stage IV) were analysed to understand the biological functions of identified ceRNAs in this study. The Gene Ontology (GO) and Kyoto Encyclopedia of Genes and Genomes (KEGG) functional enrichment analyses were conducted using the R/Bioconductor clusterProfiler (4.6.0, https://bioconductor.org/packages/release/bioc/html/clusterProfiler.html) R package^20^.

### Survival analysis

Survival analysis was performed using the Kaplan–Meier (K–M) survival curves, implemented in the survival (3.4.0, https://cran.r-project.org/web/packages/survival/index.html) R package to explore the impact of the expression level of RNAs/miRNAs on the prognostic survival of patients^21^. For each gene/lncRNA/miRNA, the tumour samples were divided into two groups (low-expressed and high-expressed) according to the median expression level. The log-rank test (Mantel–Haenszel test) was used as the statistical method for the K–M curves. The log-rank test statistic has a chi-square (χ^2^) distribution with one degree of freedom. Therefore, significant genes, lncRNAs and miRNAs were chosen under the χ^2^ test statistic p value < 0.05. The survival-significant mRNAs were checked for tumour-suppressive/oncogenic/cancer-driven roles using the CancerMine database^22^.

### Ethical approval

The study was approved by the Human Research Ethics Committees of the Queensland University of Technology (protocol code: 1900001147, date of approval: 19 December 2019) and the QIMR Berghofer Medical Research Institute (protocol code: P1051, date of approval: 23 August 2019).

## Results

### Differential expression analysis results

First, we conducted stagewise differential expression analysis to determine which genes, lncRNAs, and miRNAs are expressed at different levels between tumour and healthy groups. The number of up/downregulated lncRNAs, genes and miRNAs are available in Table 1.

**Table 1:** Counts of differentially expressed (up/down) lncRNAs, mRNAs, and miRNAs in each BC stage.

Breast cancer stage	lncRNA		mRNA		miRNA	
	Up	Down	Up	Down	Up	Down
I	50	45	1199	711	48	61
II	75	39	1385	779	62	67
III	71	43	1290	822	50	61
IV	90	43	1350	989	54	66

*lncRNA* long non-coding RNA, *mRNA* messenger RNA, *miRNA* microRNA, *Up* up-regulated,* Down* down-regulated.

The above-listed mRNAs, lncRNAs, and miRNAs were involved in the ceRNA network analysis.

### Competing endogenous RNA networks of BC stages

We constructed four ceRNA networks for BC stages I–IV. In Table 2, we have included the count of significant ceRNA associations in each BC stage. The number of lncRNAs, genes, and miRNAs involved in stage-specific ceRNA networks is given within brackets.

**Table 2:** The lncRNA-associated ceRNA networks for different breast cancer stages.

Breast cancer stage	Number of ceRNA associations (number of involved unique lncRNAs, mRNAs, and miRNAs in the network)
I	48 (2, 25, 3)
II	127 (5, 42, 55)
III	86 (5, 40, 44)
IV	142 (6, 47, 59)

*lncRNA* long-non-coding RNA, *ceRNA* competing endogenous RNA, *mRNA* messenger *RNA*, *miRNA* microRNA.

According to Table 2, stage II and IV analyses have resulted in a considerably large set of ceRNA networks compared to stages I and III.

**Figure 1:**
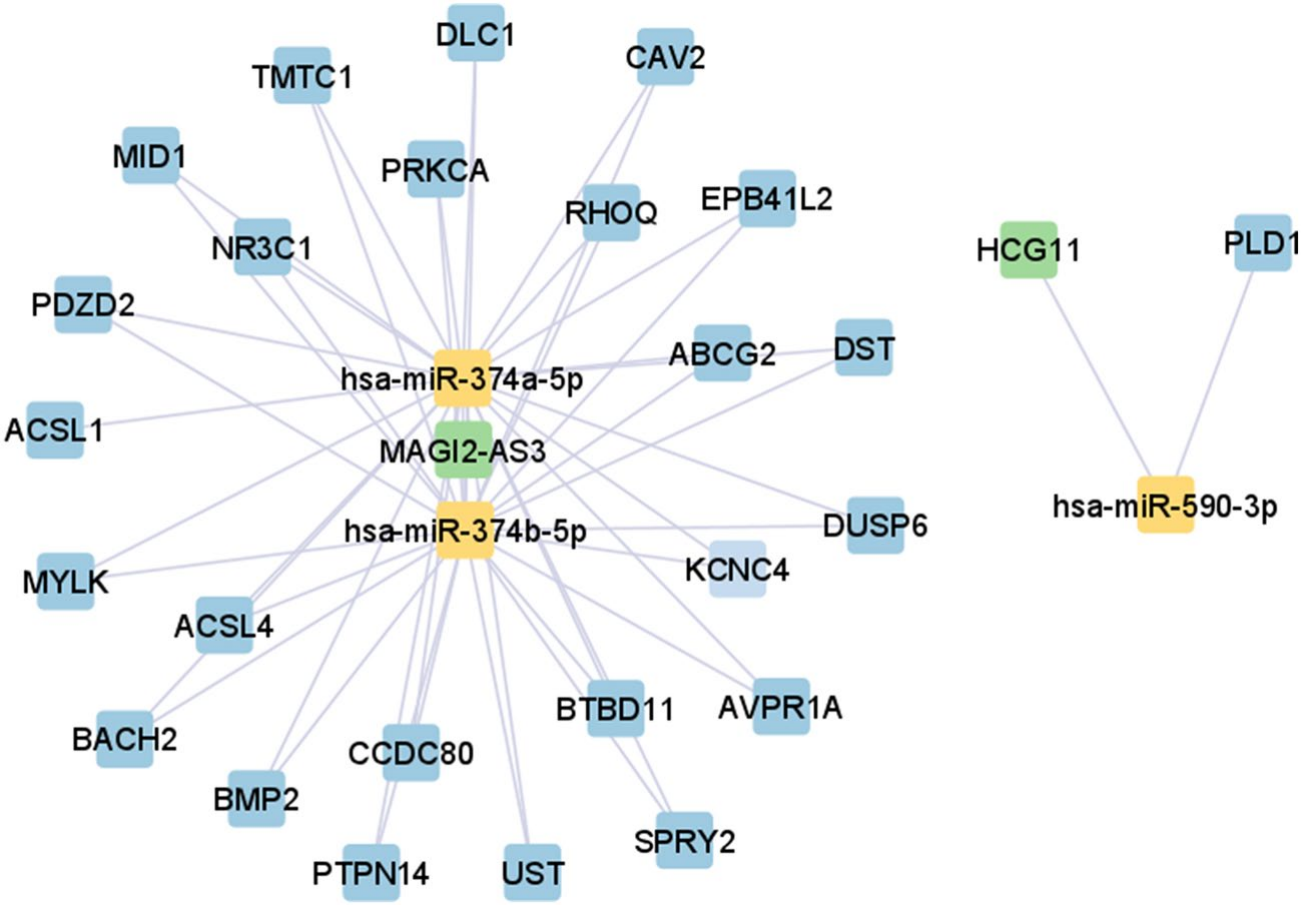
ceRNA network for stage I BC, was constructed by the Cytoscape tool^23^.

**Figure 2:**
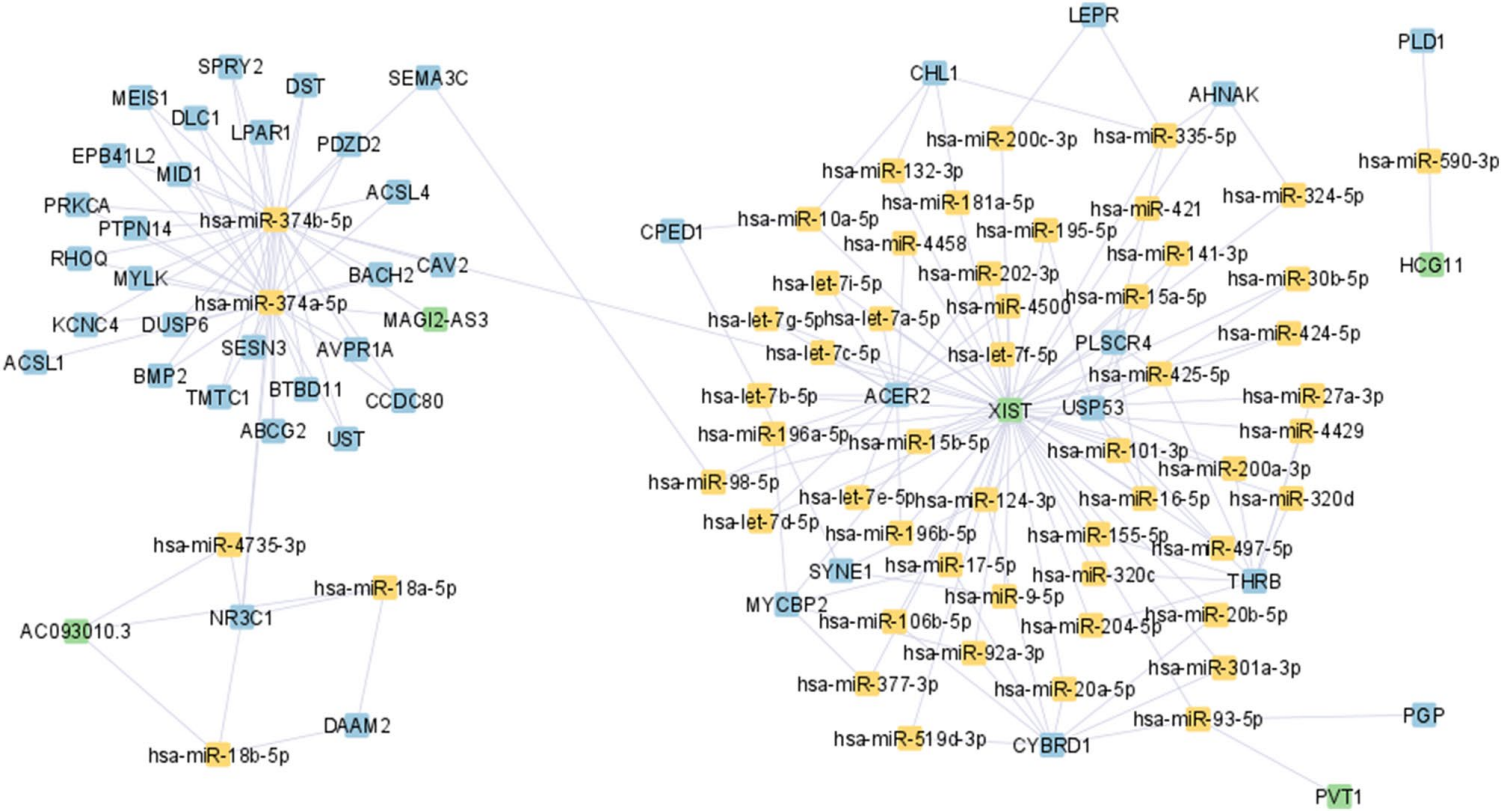
ceRNA network for stage II BC, was constructed by the Cytoscape tool^23^.

**Figure 3:**
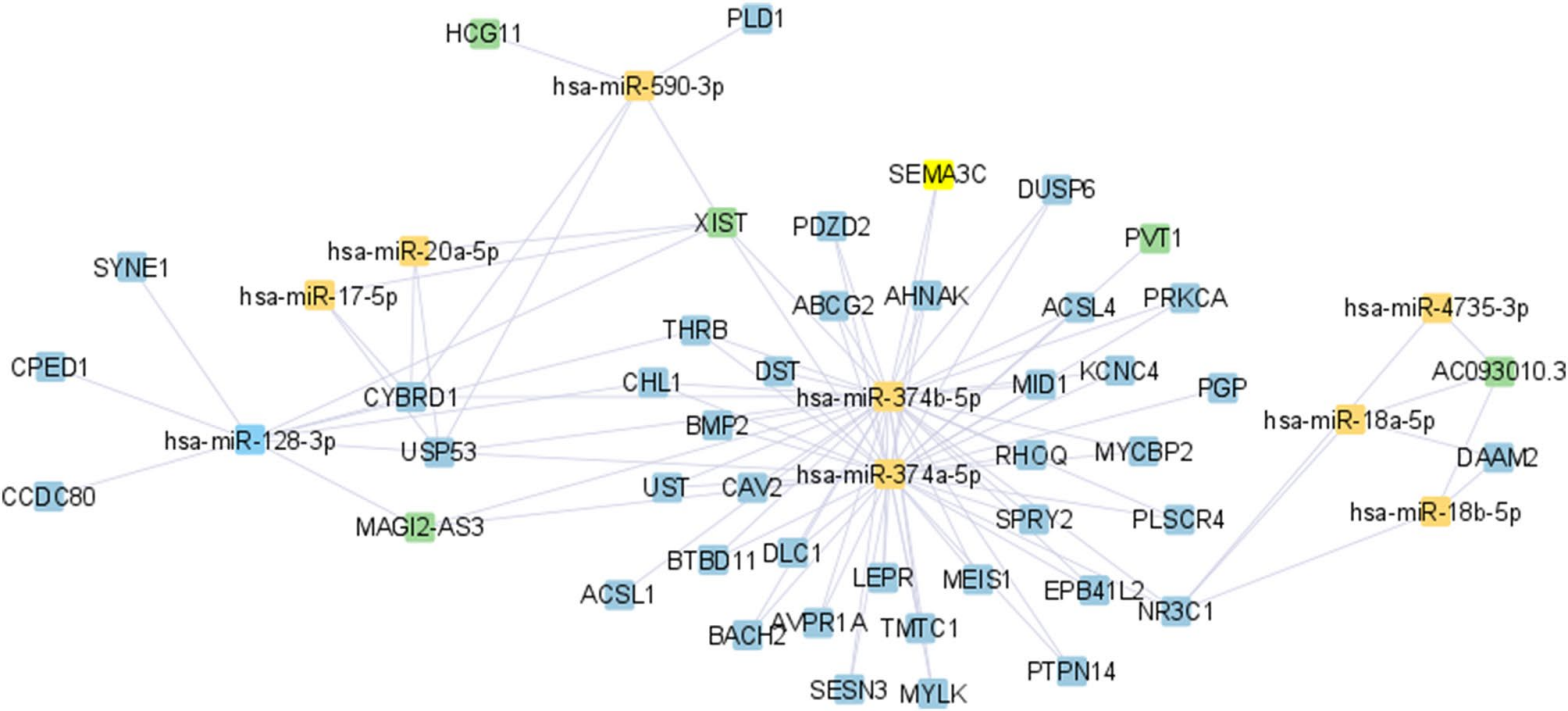
ceRNA network for stage III BC, was constructed by the Cytoscape tool^23^.

**Figure 4:**
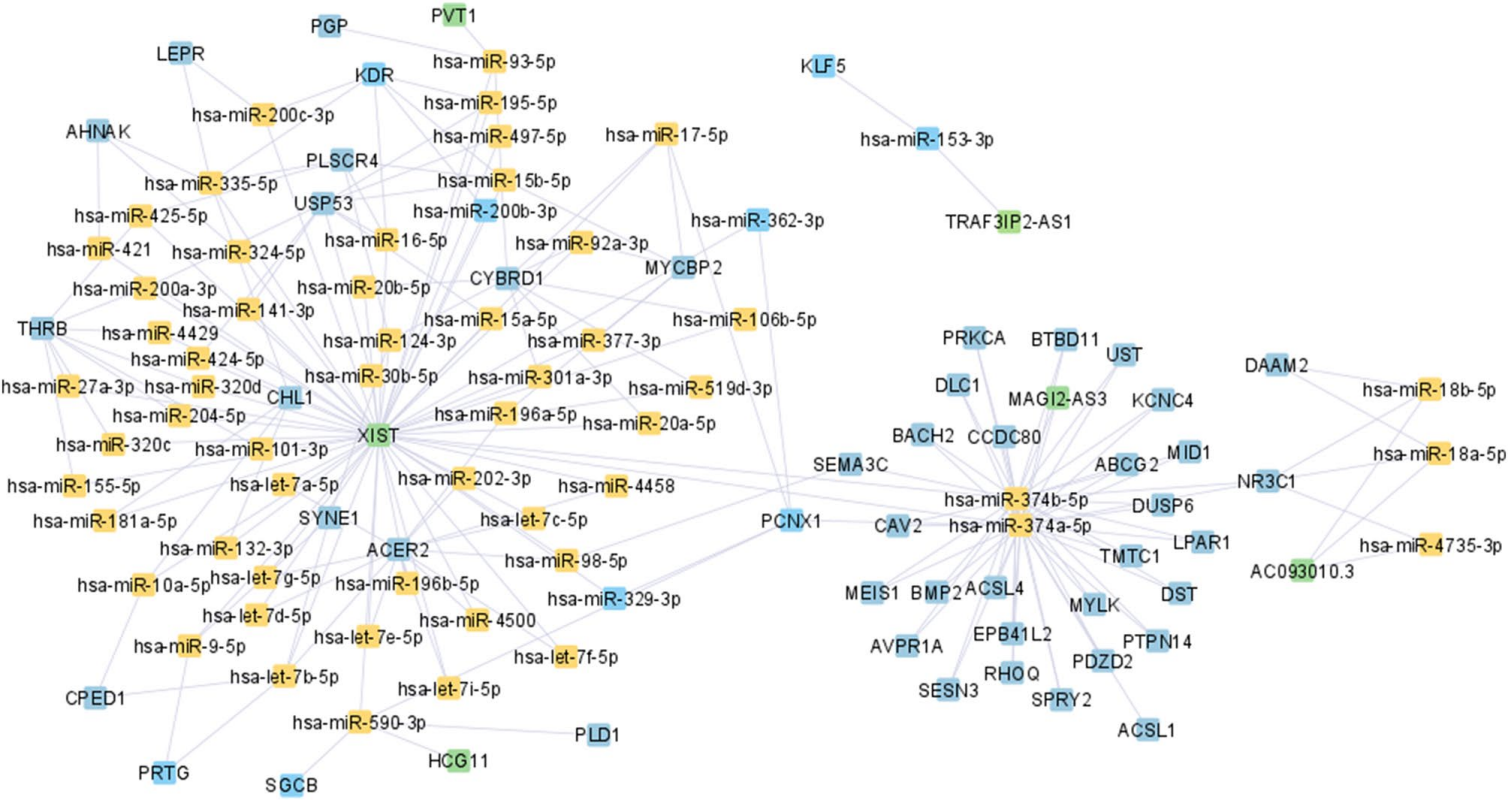
ceRNA network for stage IV BC, was constructed by the Cytoscape tool^23^.

The detailed list of significant lncRNA-mRNA-miRNA associations of each BC stage is available in Supplementary Information. The Cytoscape tool version 3.9.1 (https://cytoscape.org/download.html)^23^ was used to visualise ceRNA networks in each BC stage. Figures 1, 2, 3 and 4 illustrates ceRNA networks for BC stage I, II, II, and IV, respectively. In Figs. 1, 2, 3 and 4, blue-, green-, and yellow-coloured squares represent genes, lncRNAs, and miRNAs, respectively.

According to Figs. 1, 2, 3 and 4, hsa-miR-374a-5p and 374b-5p tend to build up separated ceRNA clusters in each BC stage. As shown in Fig. 4, the KLF5 gene and TRAF3IP2-AS1 lncRNA, unique to stage IV, create a unique triplet with hsa-miR-153-3p.

**Figure 5:**
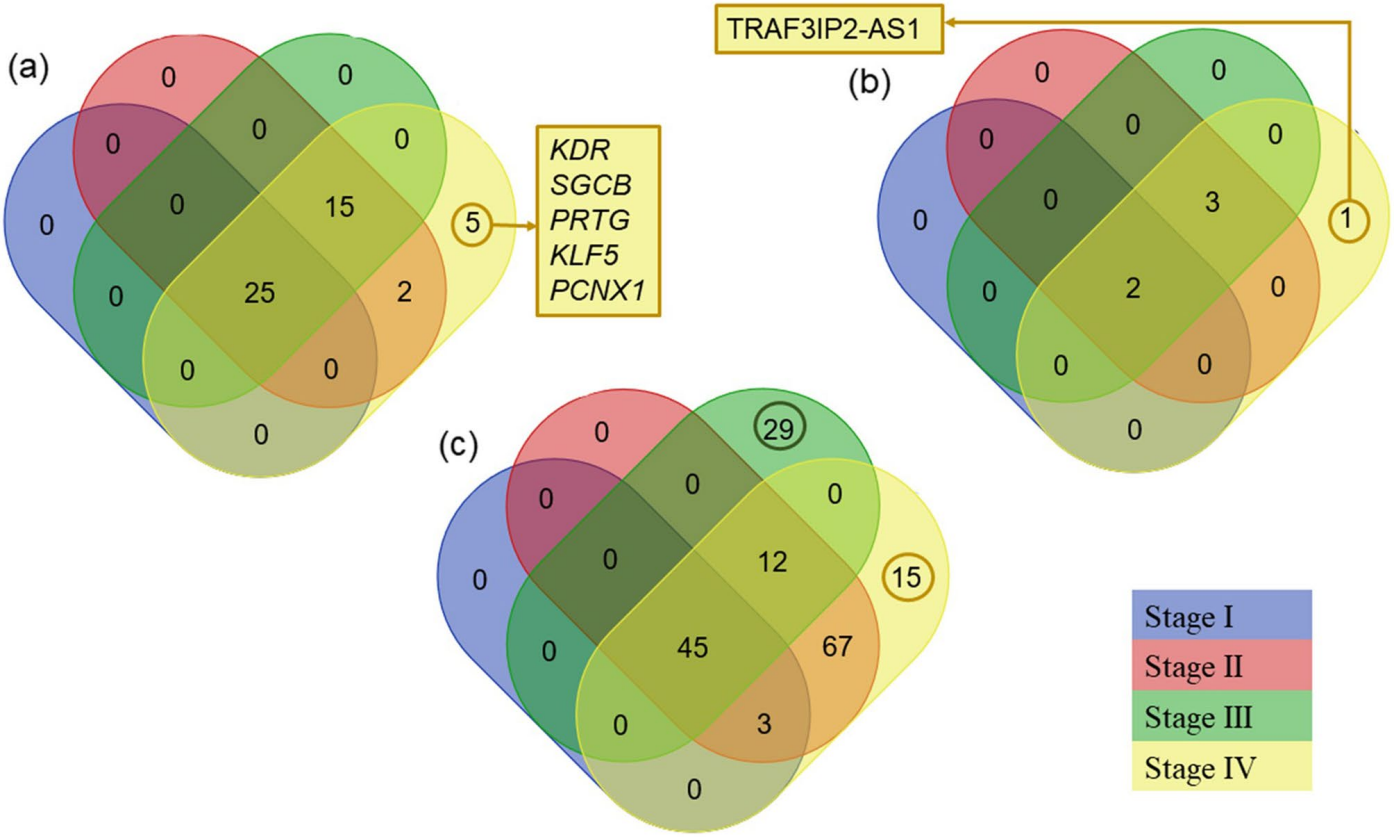
Venn diagram representation for mRNAs (**a**), lncRNAs (**b**), and competing endogenous RNAs (ceRNAs) (**c**) included in stage-specific significant ceRNA associations. Blue, Red, green, and yellow-coloured sets represent stage I, stage II, stage III, and stage IV, respectively. Most of the mRNAs and lncRNAs have been shared among four stages. Five genes, *KDR*, *SGCB*, *PRTG*, *KLF5*, and *PCNX1* and one lncRNA, TRAF3IP2-AS1, were observed only in stage IV, metastatic stage.

The lncRNAs, genes and miRNAs list of significant ceRNA associations were combined for a single variable as "<lncRNA gene ensemble ID>_<gene ensemble ID>_<miRNA name>" (Ex: ENSG00000234456_ENSG00000125845_hsa-miR-374b-5p). The values of gene ensemble ID, lncRNA ensemble ID, and the derived variable columns were inserted into a four-set Venn diagram representation (indicating four BC stages) as Fig. 5a–c, respectively.

According to Fig. 5, most lncRNAs, mRNAs, and lncRNA-mRNA-miRNA associations found in each BC stage ceRNA network have been shared among more than one stage. One lncRNA (TRAF3IP2-AS1) and five mRNAs (*KDR*, *SGCB*, *PRTG*, *KLF5*, and *PCNX1*) have been observed only in the stage-IV-specific ceRNA network. We identified 29 and 15 unique ceRNA associations in stage-III and stage-IV BC, respectively.

### Functional enrichment analysis for stage-specific competing endogenous RNA networks

**Figure 6:**
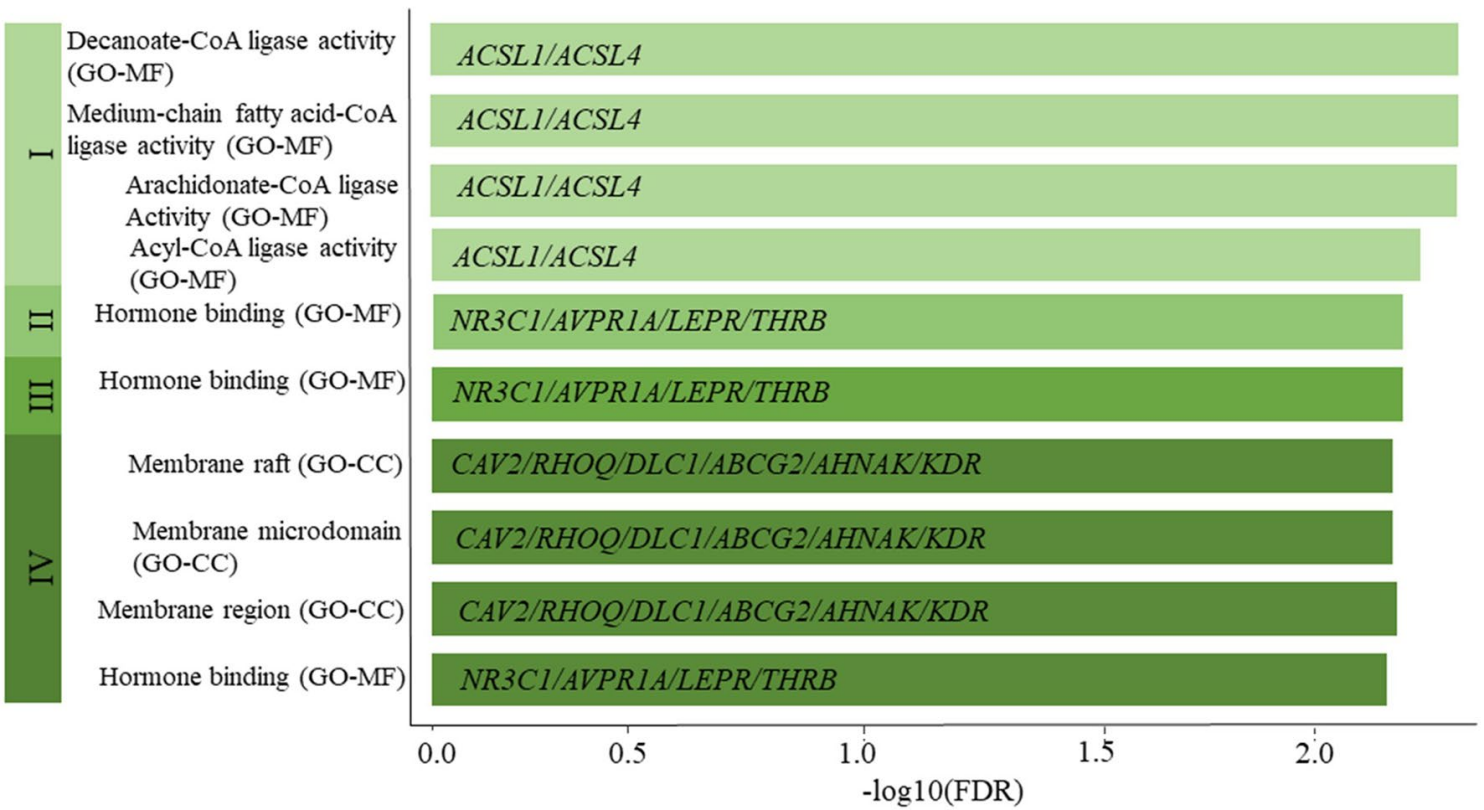
Pathway enrichment analysis results for genes included in stage-specific competing endogenous RNA networks in breast cancer^20^. Four, one, one, and four gene-ontology pathways were statistically significant in stages I, II, III, and IV, respectively. The CoA ligase activity-related pathways have been significant only in stage I. The hormone-binding pathway was significant among stages II, III, and IV.

We conducted functional enrichment analyses on 25, 42, 40, and 47 genes obtained from the stage I, stage II, stage III, and stage IV ceRNA networks. None of the stage-specific genes was enriched in KEGG pathways^24^. Two genes resulting from stage I analysis were enriched in four CoA ligase activity-associated GO-molecular function (GO-MF) pathways. The GO-MF hormone-binding pathway was significant across stages II, III, and IV. In stage IV, six genes were enriched in three GO-cellular components (CC) pathways, membrane raft, membrane microdomain, and membrane region. Figure 6 illustrates GO pathway results for each BC stage.

### Survival analysis for stage-specific competing endogenous RNA networks

**Figure 7:**
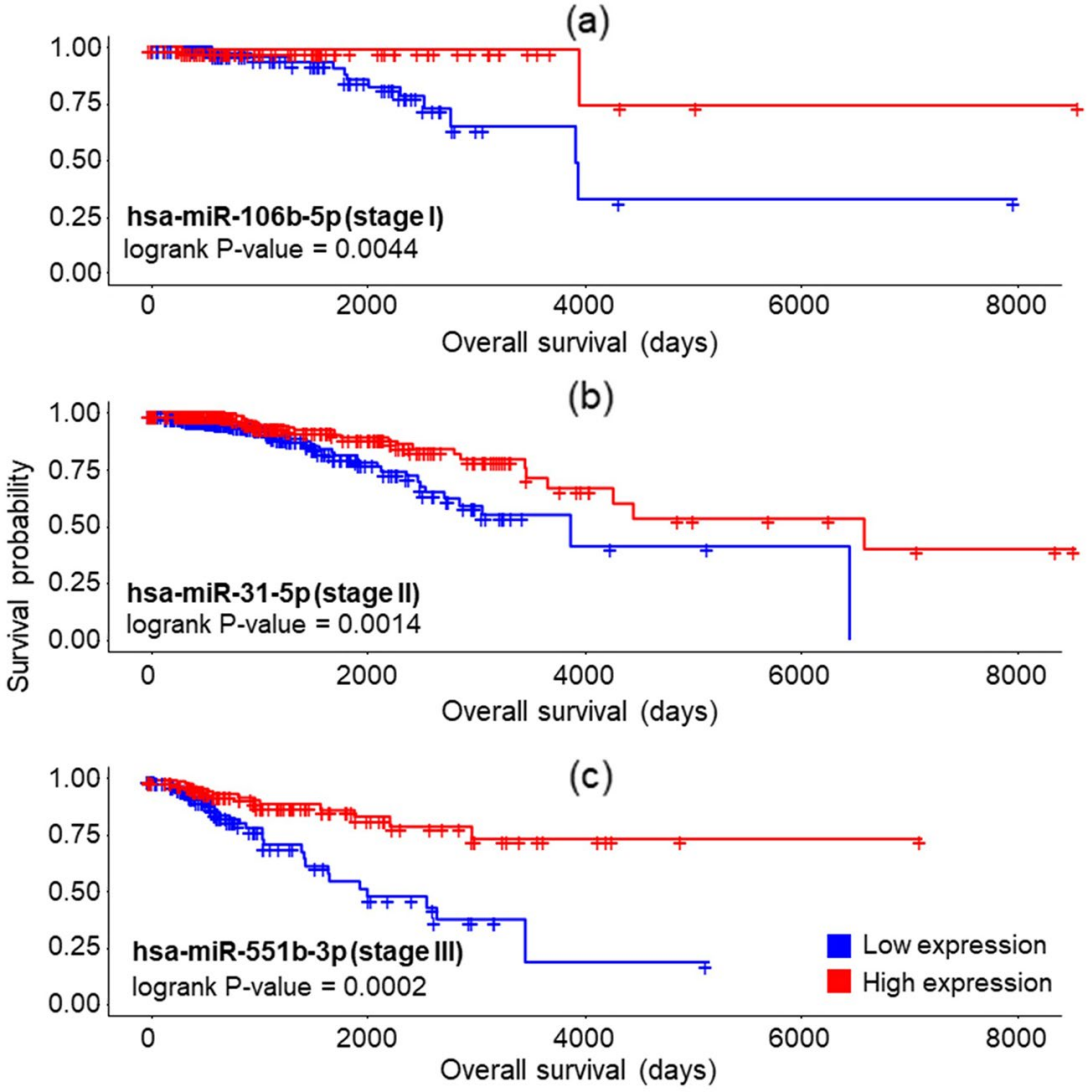
Kaplan–Meier survival plots for the top significant miRNAs in stage-specific breast cancer networks^21^. The high-expressed hsa-miR-106b-5p (**a**), hsa-miR-31-5p (**b**), and hsa-miR-551b-3p (**c**) were prognostic in stage I, II, and III, respectively. None of the microRNAs in the stage IV ceRNA network was significant from survival analysis.

K-M survival analyses and log-rank tests were performed to identify the potential stage-specific differentially expressed genes, lncRNAs and miRNAs strongly correlated with BC patients' prognostic characteristics. The significant genes, lncRNAs and miRNAs, were chosen under p value < 0.05. We found two, three, three, and one gene(s) as prognostic biomarkers in stage I, stage II, stage III, and stage IV, respectively and described in Table 3. None of the lncRNAs was identified as biomarkers in stage-specific BC survival analysis. In miRNA-based survival analyses, two, three, and three miRNAs were statistically significant in stage I, stage II, and III, respectively. Figure 7 illustrates K–M curves for the top significant miRNA of each stage, hsa-miR-106b-5p in stage I, hsa-miR-31-5p in stage II, and hsa-miR-551b-3p in stage III.

**Table 3:** Statistically significant genes from stage-specific survival analyses.

Stage	Gene	HR	p-value
I	*BMP2*	0.2934	0.0233
I	*BACH2*	0.2817	0.0185
II	*DUSP6*	0.5397	0.0122
II	*ACSL1*	1.8924	0.0111
II	*MYCBP2*	1.6608	0.0388
III	*DST*	0.5074	0.0234
III	*PDZD2*	1.9969	0.0291
III	*AHNAK*	0.2718	0.0221
IV	*MID1*	0.0895	0.0143

*HR* hazard ratio.

## Discussion

This study identified stage-specific lncRNA-mRNA-miRNA ceRNA associations in BC. According to ceRNA network analysis results, most ceRNA associations were shared across all four stages. In contrast, one lncRNA (TRAF3IP2-AS1) and five genes (*KDR*, *PRTG*, *KLF5*, *SGCB*, and *PCNX1*) were statistically significant only in metastatic BC, i.e., stage IV. The lncRNA TRAF3IP2-AS1 has been previously reported in renal cell carcinoma and glioblastoma but not in BC^25^.

According to AACR (American Association for Cancer Research) project GENIE (Genomics Evidence Neoplasia Information Exchange), the *KDR* gene is altered in 1.54% of BC patients. It can play an essential role in mediating endothelial cells proliferation, migration, and permeability^26^. Endothelial cells are actively involved in cancer metastasis^27^. The *PRTG* gene has not been previously described in BC. Nevertheless, *PRTG* has been identified as an oncogenic protein in gastric carcinogenesis by activating the downstream cGMP/PKG signalling pathway^28^.

The *KLF5* gene is found to play a role in BC, but its precise function remains determined. On the one hand, the *KLF5* locus at chromosome 13 is frequently deleted in human BC, and its protein is degraded by the WWP1 oncogenic ubiquitin E3 ligase, which suggests a tumour-suppressor function^29^. On the other hand, increased expression of *KLF5* is associated with expression of the HER2 oncoprotein and shorter survival in BC patients suggesting an oncogenic function of *KLF5* in BC^30^. A recent bioinformatic study has found that SGCB protein is specific in basal A subtyped BC gene regulatory networks^31^. The *PCNX1* has been a potential marker of response to chemotherapy in BC, and therapeutic modulation of its activities could enhance chemotherapy responses^32^. The BC studies mentioned above have described four out of five genes found from our stage IV-specific ceRNA network, and none of these studies explains their contribution to metastatic BC. Therefore, wet-lab experiments will be carried out in the future to investigate their role in BC metastatic nature.

Two downstream analyses, functional enrichment analysis and survival analysis, have ensured stage-specific ceRNA components found in our study. All statistically significant genes resulting from stagewise survival analyses have been previously reported in cancer studies^22^. *BMP2* from the stage I survival analysis has shown oncogenic function in BC^33^. Only *DUSP6* and *ACSL1* in stage II have acted as an oncogene and a tumour suppressor in BC, respectively^34, 35^. *DST* and *AHNAK* genes that were significant from the stage III survival analysis have shown tumour suppressive characteristics in both experimental and computational BC studies^36–40^. In stage IV, we identified *MID1* as the only survival significant gene and it has been linked with invasive lobular carcinoma^22^. The invasive lobular carcinoma is the second most common type of BC. It originates in the milk-producing gland (lobules) of the breast. Invasive cancer is recognised as the cancer cells have broken out of the lobules where they initiated and are potential to expand to the lymph nodes and other areas of the body, leading to metastasis^41^. Therefore, *MID1* gene should be further investigated to identify its role in metastatic BC (stage IV).

We found eight miRNAs from the stage-specific survival analyses, and these miRNAs have shown a tumour-suppressive/oncogenic role in previous BC experimental studies. The hsa-miR-106b-5p and hsa-miR-374b-5p were associated with the prognosis of stage I BC patients. The hsa-miR-106b-5p promoted cell migration, invasion, and proliferation by targeting FUT6^42^. Abnormal hsa-miR-374b-5p expression in luminal-HER2-positive BC cells can be used for classifying clinicopathologic subtypes of BC^43^. Three miRNAs, hsa-miR-150-5p and hsa-miR-31-5p, are significant in stage II BC survival analysis, and hsa-miR-374b-5p was significant in both stages I and II. The other two miRNAs, hsa-miR-150-5p and hsa-miR-31-5p, have shown an oncogenic and tumour-suppressive role in BC, respectively^44,45^. We identified three miRNA signatures (hsa-miR-551b-3p, hsa-miR-101-3p, and hsa-miR-26a-5p) as prognostics in stage III BC survival. Among them, hsa-miR-551b-3p have promoted oncogenic features in BC cells^46^. In previous BC experimental studies, both hsa-miR-101-3p and hsa-miR-26a-5p have shown a tumour-suppressive role^47,48^. We did not find statistically significant prognostic miRNAs from the stage IV BC survival analysis.

Functional enrichment analyses found molecular pathways associated with protein-coding genes in stage-specific ceRNA networks. Two genes in Acyl-CoA Synthetase Long (ACSL) Chain family, *ACSL1* and *ACSL4*, included only in stage I ceRNA networks, are enriched in four CoA ligase activity-associated pathways. Therefore, wet-lab experiments are required to understand the tumour-suppressive/oncogenic/cancer-driven role of ACSL Chain family members among early-stage BC patients. Four genes found in stages II, III, and IV (*NR3C1*, *AVPR1A*, *LEPR*, and *THRB*) are associated with hormone binding, which plays a role in BC pathophysiology and defining risk. Six genes in the stage IV ceRNA network are enriched in three components in the GO-CC pathway: membrane raft, membrane microdomain, and membrane region. These membrane domains have shown an important role in cancer metastasis^49^.

Our study has shared a limited set of ceRNAs with the previous ceRNA network study for overall BC cases by Tuersong et al.^3^. Four miRNAs (hsa-miR-141, hsa-miR-200a, hsa-miR-204, and hsa-miR-301b) have been identified in ceRNA networks in both studies. Among these four miRNAs, Tuersong et al.^3^ have demonstrated that hsa-miR-204 was downregulated and hsa-miR-301b was upregulated in patients with BRCA compared with healthy controls and were associated with overall survival. Our previous transcriptome-wide association study also demonstrated hsa-miR-204 as a tumour-suppressive miRNA in prostate cancer with statistically significant low expressed levels in prostate cancer cell lines^50^. Moreover, we found two genes, *SPRY2* and *CHL1*, involved in Tuersong et al.^3^ and our works (*SPRY2* in stages I–IV and *CHL1* in stages II, III, and IV). Observing a smaller number of shared ceRNAs between studies can be occurred due to higher heterogeneity between breast cancer stages, and it will lead to different RNA/gene expression levels. Zhou et al. conducted a ceRNA network analysis on BRCA subtypes, basal-like, HER2+, luminal A, and luminal B^51^. The authors have identified three lncRNAs, NEAT1, OPI5-AS1, and AC008124.1, among all four subtype-related ceRNA networks. Moreover, three lncRNAs, NEAT1, FAM83H-AS1, and XIST1, were significantly differentially expressed in the basal-like subtype-related network. Nevertheless, we could not find a shared outcome between our study and subtype-related networks. This can be due to our stage-based analyses containing RNA/gene expression levels from multiple subtypes.

This study is limited to ceRNA networks mediated by microRNA expression levels. Other genomic (copy number alteration), transcriptomic (transcription factors), and epigenetic (DNA methylation) factors were not considered in the ceRNA network analysis^52^. Moreover, other possible ceRNA components such as pseudogenes and lincRNAs were not considered. Therefore, future studies should be extended to address these concerns. Nevertheless, this study elucidates a new level of ceRNA network analysis, stage-specific ceRNA networks, to understand better common/unique ceRNA(s) among/within the stage(s) of a given cancer. Identifying novel stage-level cancer biomarkers will significantly contribute to the knowledge of personalised therapeutics and determining risk.

## Conclusions

We conducted ceRNA networks analyses in four stages of BC. Only one lncRNA and five genes were significant in the stage IV BC ceRNA network. Further validation experiments are required to characterise their role in BC metastatic nature. Identifying ceRNA components across cancer stages will advance the diagnosis, risk identification, and therapeutics.

## Supplementary Information

Supplementary Information.

## Data Availability

Publicly available TCGA BC RNA-seq and miRNA-seq expression data were downloaded through the GDC Data Portal (https://portal.gdc.cancer.gov/repository). All statistical analyses and graph preparations were performed using the R statistical software, freely available at https://cran.r-project.org/. The datasets used and/or analysed during the current study available from the corresponding author on reasonable request.
